# Association Between MGMT Promoter Methylation and Clinical and Lifestyle Factors in Glioblastoma: A Single-Center Study in Korea

**DOI:** 10.3390/jcm15031305

**Published:** 2026-02-06

**Authors:** Mee-Seon Kim, Yu-Mi Lee, Shin-Ah Son, DongJa Kim, Chaejin Lee, Jeong-Hyun Hwang

**Affiliations:** 1 Department of Pathology, School of Dentistry, Kyungpook National University Hospital, Kyungpook National University, Daegu 41944, Republic of Korea; meeseonkim@knu.ac.kr; 2 Department of Preventive Medicine, School of Medicine, Kyungpook National University, Daegu 41566, Republic of Korea; 3 Trauma Center, Department of Thoracic and Cardiovascular Surgery, School of Medicine, Kyungpook National University Hospital, Kyungpook National University, Daegu 41944, Republic of Korea; 4 Department of Forensic Medicine, School of Medicine, Kyungpook National University Hospital, Kyungpook National University, Daegu 41944, Republic of Korea; 5 Department of Neurosurgery, School of Medicine, Kyungpook National University Hospital, Kyungpook National University, Daegu 41944, Republic of Korea

**Keywords:** glioblastoma, MGMT promoter methylation, smoking, hypertension, epigenetics

## Abstract

**Background/Objectives:** Although O6-methylguanine-DNA methyltransferase (MGMT) promoter methylation is a key predictive biomarker in glioblastoma, its association with clinical and lifestyle characteristics remains poorly understood. **Methods:** We retrospectively analyzed 105 patients who underwent surgical treatment for glioblastoma at Kyungpook National University Hospital between August 2012 and April 2022 to evaluate the relationship between MGMT promoter methylation status and clinical and lifestyle factors. Collected variables included age, sex, body weight, body height, smoking history, and comorbidities such as hypertension, diabetes mellitus, and hyperlipidemia. **Results:** Current smoking was significantly associated with MGMT promoter methylation in both univariate and multivariate analyses (adjusted odds ratio [OR], 4.6; *p* = 0.03). Additionally, a history of hypertension was associated with MGMT promoter methylation after multivariate adjustment (adjusted OR, 3.6; *p* = 0.03). **Conclusions:** MGMT promoter methylation in glioblastoma was associated with current smoking and a history of hypertension, suggesting lifestyle-related factors may influence epigenetic mechanisms underlying MGMT promoter methylation in glioblastoma.

## 1. Introduction

Glioblastoma is the most common malignant primary brain tumor in adults and is associated with a dismal prognosis [1,2]. The current standard of care, consisting of maximal safe resection followed by radiotherapy with concomitant and adjuvant temozolomide, has improved survival; however, the prognosis remains poor, with a median survival of approximately 15 months [3,4]. Among established prognostic biomarkers, promoter methylation of the O6-methylguanine-DNA methyltransferase (MGMT) gene is a key determinant of response to temozolomide and an important prognostic factor in glioblastoma [5]. Temozolomide (TMZ) is an orally administered alkylating agent with good permeability across the blood–brain barrier, which underlies its therapeutic efficacy in brain tumors, including glioblastoma [4,6,7,8]. MGMT repairs O6-methylguanine DNA lesions; promoter methylation leads to epigenetic silencing of the MGMT gene, resulting in impaired repair of temozolomide-induced DNA damage and enhanced treatment response [5,7,8]. Accordingly, combined radiotherapy and temozolomide chemotherapy have resulted in a modest prolongation of survival [5,9]. This implies a role for MGMT as a target for radiosensitization [10] and reiterates the importance of research on staging radiosurgery [11] and the use of adjuvant radiosensitizers [12] in glioblastoma patients. While MGMT promoter methylation is a key predictive biomarker in glioblastoma, little is known about its association with clinical characteristics such as age, sex, smoking history, body mass index (BMI) and comorbidities. Although previous studies have investigated the relationship between MGMT promoter methylation and clinical characteristics in glioblastoma, they have generally focused on a limited range of variables [13,14]. To date, the relationship between MGMT promoter methylation and lifestyle-related factors, including smoking, obesity, and comorbidities, has not been extensively explored. Smoking and obesity have been suggested to be associated with epigenetic dysregulation and systemic inflammation [15,16,17,18,19]. Moreover, comorbid conditions such as hypertension and diabetes mellitus may be associated with epigenetic alterations, including changes in DNA methylation patterns [17,20,21]. Given that glioblastoma can be broadly classified into MGMT-methylated and unmethylated subgroups, it remains unclear whether differences in lifestyle factors, comorbid conditions, or obesity exist between these two groups. A clearer understanding of these differences may contribute to improved interpretation of MGMT promoter methylation status and provide insights into the epigenetic heterogeneity of glioblastoma. Therefore, we conducted this study to investigate potential differences in clinical and lifestyle characteristics according to MGMT promoter methylation status in patients with glioblastoma. Exploring these relationships may provide insight into the epigenetic heterogeneity of glioblastoma and improve the clinical interpretation of MGMT status. The aim of this study is to evaluate the relationship between MGMT promoter methylation status and clinical and lifestyle factors, including sex, age, BMI, and smoking history, in patients with glioblastoma.

## 2. Materials and Methods

We retrospectively reviewed the electronic medical records of 128 patients who underwent surgical treatment for glioblastoma at the Department of Neurosurgery, Kyungpook National University Hospital, between August 2012 and April 2022. Out of the initial 128 patients, 13 cases were excluded due to insufficient information in the medical records, and 10 cases were excluded because the specimens were inadequate for a definitive diagnosis, leaving a total of 105 cases for analysis. The clinical data collected included MGMT promoter methylation status, age, sex, body weight, height, smoking history, and medical histories of hypertension, diabetes mellitus, and hyperlipidemia. Information regarding dietary habits was not available from the medical records. Therefore, dietary habits were not included as a variable among lifestyle factors. All patients included in the study had no previous history of brain surgery or temozolomide (TMZ) treatment at the time of enrollment.

**Figure 1:**
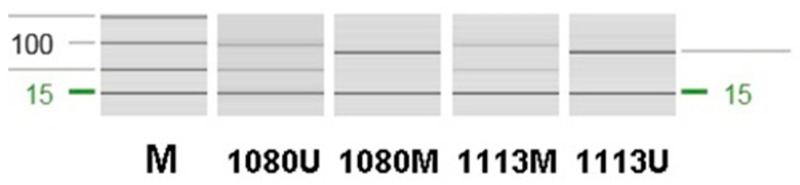
Representative capillary electrophoresis profiles of methylation-specific PCR (MSP) analysis for the MGMT promoter. Lane M indicates the molecular size marker. Unmethylated (U) and methylated (M) MGMT promoter–specific bands are shown. The expected sizes of the unmethylated and methylated PCR products are 93 bp and 81 bp, respectively.

MGMT promoter methylation status was determined based on results documented in the electronic medical records, which were originally obtained by methylation-specific polymerase chain reaction (MSP) analysis of DNA extracted from postoperative formalin-fixed, paraffin-embedded (FFPE) tumor tissues. Methylation-specific PCR (MSP) was performed using an EpiTect MSP Kit (Qiagen, Hilden, Germany) according to the manufacturer’s protocol. Primer sequences are listed in Table 1. Reactions were prepared in a total volume of 25 μL and amplified on a TPersonal Thermal Cycler (Biometra GmbH, Göttingen, Germany) under the following conditions: 95 °C for 10 min; 40 cycles of 95 °C for 30 s, 62 °C for 30 s, and 72 °C for 30 s; and a final extension at 72 °C for 10 min. PCR products were analyzed by capillary electrophoresis using the QIAxcel Advanced system (Qiagen). The unmethylated and methylated products were 93 bp and 81 bp in size, respectively (Figure 1). This retrospective study was approved by the Institutional Review Board of Kyungpook National University Hospital (IRB No. 2022-05-009; approved on 13 June 2022). The requirement for informed consent was waived because the study involved retrospective review of existing medical records and posed minimal risk to participants.

### 2.1. Histopathological and MGMT Methylation Review

Histopathological slides and MGMT promoter methylation results were independently reviewed by two board-certified pathologists (Ms-K and Dj-K) to confirm the diagnosis; cases in which the specimens were inadequate for a definitive diagnosis were excluded from the analysis. Histological slides were reviewed and interpreted according to the World Health Organization (WHO) classification of tumors of the central nervous system, 5th edition [22,23]. The reviewed diagnosis was concordant with the original diagnosis. MGMT promoter methylation status was classified as methylated when a methylated-specific band was detected, regardless of the presence of an unmethylated band. Cases showing only unmethylated-specific amplification were classified as unmethylated. Sixteen patients underwent two or more surgical resections due to tumor recurrence, and MGMT promoter methylation analysis was performed at each surgical intervention (two resections in 14 patients, three resections in one patient, and four resections in one patient). In all 16 patients, the MGMT promoter methylation status was concordant between the primary tumor and all subsequent recurrent tumors.

### 2.2. Definition of Clinical and Lifestyle Variables

Body mass index (BMI) was calculated as body weight in kilograms divided by the square of height in meters (kg/m^2^). Height and weight were obtained from medical records at the time of diagnosis (or at initial evaluation). The BMI classification was based on the Asia-Pacific BMI Classification, which recommends lower BMI cutoffs for defining overweight and obesity in Asian populations due to differences in body composition and metabolic risk compared with Western populations. A BMI of 18.5–22.9 kg/m^2^ was classified as normal, 23.0–24.9 kg/m^2^ as overweight, and ≥25.0 kg/m^2^ as obese [24]. Smoking status was classified as current smoker, former smoker, or never smoker. A current smoker was defined as a patient who actively smoked at the time of glioblastoma diagnosis, whereas a former smoker was defined as a patient who had quit smoking prior to diagnosis. Never smokers were defined as patients who had never smoked. Hypertension was defined as a documented diagnosis of hypertension and/or ongoing treatment with antihypertensive medication at the time of glioblastoma diagnosis. Diabetes mellitus was defined as a documented diagnosis of diabetes mellitus and/or ongoing treatment with antidiabetic medication at the time of diagnosis. Among the included patients, all cases of diabetes mellitus were classified as type 2 diabetes, and no patients with type 1 diabetes were included.

### 2.3. Statistical Analysis

Statistical analyses were performed using R software (version 4.3.1; R Foundation for Statistical Computing, Vienna, Austria). The Hmisc and survival packages were used for data processing and analysis. For univariate analyses, the Wilcoxon rank-sum test (Table 2) and Fisher’s exact test (Table 2 and Table 4) were applied as appropriate. Multivariate analyses were conducted using a logistic regression model (Table 3). Statistical significance was defined as a *p*-value < 0.05.

## 3. Results

### 3.1. Study Population

The patients’ ages ranged from 41 to 87 years (median, 65 years). There were 57 male and 48 female patients, yielding a male-to-female ratio of 1.19:1. The body mass index (BMI), calculated from recorded height and weight, ranged from 18.8 to 31.3 kg/m^2^ (mean, 23.8 kg/m^2^; median, 23.6 kg/m^2^). Seventy-one patients were classified as having normal weight, while 34 patients were classified as overweight or obese. With regard to smoking status, 15 patients were current smokers, 18 were former smokers, and 72 were never smokers. Among ever smokers, pack-year exposure ranged from 10 to 100 pack-years (mean, 33.15; median, 30).

### 3.2. Univariate Analysis of Factors Associated with MGMT Promoter Methylation

In the univariate analysis, MGMT promoter methylation was observed at a significantly higher frequency in current smokers compared with former and never smokers (*p* = 0.04; Table 2). No other variables showed statistically significant associations in the univariate analysis. These findings are consistent with those reported in previously published studies [25], in which MGMT promoter methylation was more frequently observed in smokers than in never-smokers among patients with non–small cell lung cancer.

**Table 2:** Univariate Analysis According to MGMT Promoter Methylation Status.

Variable	MGMT (+) (n = 48)	MGMT (−) (n = 57)	*p*-Value
**Age, years (mean)**	41–84 (64.7)	47–87 (65.2)	0.8
**Sex, n (%)**			0.6
Male	28 (58.3)	29 (50.9)	
Female	20 (41.7)	28 (49.1)	
**Body mass index (BMI)** **, kg/m^2^ (median)**	18.8–31.3 (23.6)	18.8–31.2 (23.6)	0.8
**Weight category, n (%)**			0.3
Normal weight	35 (72.9)	36 (63.2)	
Overweight/Obese	13 (27.1)	21 (36.8)	
**Smoking status, n (%)**			**0.04**
Never or former smoker	37 (77.1)	53 (93.0)	
Current smoker	11 (22.9)	4 (7.0)	
**Hypertension, n (%)**			0.3
Yes	19 (39.6)	17 (29.8)	
No	29 (60.4)	40 (70.2)	
**Type 2 diabetes, n (%)**			0.5
Yes	4 (8.3)	8 (14.0)	
No	44 (91.7)	49 (86.0)	
**Dyslipidemia, n (%)**			0.6
Yes	5 (10.4)	9 (15.8)	
No	43 (89.6)	48 (84.2)	

MGMT (+), positive for MGMT promoter methylation; MGMT (−), negative for MGMT promoter methylation.

### 3.3. Multivariate Analysis of Factors Associated with MGMT Promoter Methylation

In multivariate logistic regression analysis, consistent with the univariate findings, current smoking remained significantly associated with an increased likelihood of MGMT promoter methylation (odds ratio [OR], 4.6; *p* = 0.03; Table 3). Smoking showed a significant association with MGMT promoter methylation in both univariate and multivariate analyses, suggesting that this relationship is not solely attributable to confounding factors. The consistency of our findings with previous reports further supports the reliability of this association [26]. Although a history of hypertension was more frequently observed in the MGMT promoter–methylated group, this association did not reach statistical significance in the univariate analysis. However, after adjustment for potential confounders in the multivariate model, a history of hypertension showed a significant association with MGMT promoter methylation (OR, 3.6; *p* = 0.03; Table 3). To our knowledge, no previous studies have specifically examined the association between hypertension and MGMT promoter methylation. Therefore, the observed association in our study represents a novel finding.

**Table 3:** Multivariate Logistic Regression Analysis of Factors Associated with MGMT Promoter Methylation in Patients with Glioblastoma.

Variable	Odds Ratio	95% CI	*p*-Value
Age (per year)	1	0.93–1.03	0.4
Female sex	1.1	0.43–2.62	0.9
Overweight or obese	0.6	0.23–1.49	0.3
**Current smoker**	**4.6**	**1.21–20.13**	**0.03**
**Hypertension**	**3.6**	**1.15–11.95**	**0.03**
Type 2 diabetes mellitus	0.3	0.05–1.42	0.1
Dyslipidemia	0.5	0.12–1.92	0.3

Odds ratios (ORs) and 95% confidence intervals (CIs) were estimated using multivariate logistic regression.

### 3.4. Association Between Smoking Exposure and MGMT Promoter Methylation

The association between smoking exposure, quantified by pack-years, and MGMT promoter methylation was further evaluated. Among patients negative for MGMT promoter methylation, pack-year exposure ranged from 10 to 100 pack-years (median, 22.5; mean, 32.7), whereas in the MGMT promoter–methylated group, pack-year exposure ranged from 13 to 50 pack-years (median, 30; mean, 33.5). When pack-year exposure was analyzed as a continuous variable, no statistically significant association was observed between pack-years and MGMT promoter methylation, either among ever smokers (*p* = 0.25) or current smokers (*p* = 0.26). However, based on thresholds used in previous studies [27,28], ever smokers were further stratified according to smoking exposure (<20 pack-years vs. ≥20 pack-years), and this categorical analysis demonstrated a statistically significant association with MGMT promoter methylation (odds ratio [OR], 6; *p* = 0.047; 95% confidence interval [CI], 0.83–73.47; Table 4). Notably, the wide confidence interval suggests limited precision of the estimated effect size, likely reflecting the relatively small sample size of ever smokers in this subgroup analysis, and therefore these findings should be interpreted with caution.

**Table 4:** Association Between Smoking Exposure (Pack-Years) and MGMT Promoter Methylation Among Ever Smokers.

	<20 Pack-Years (n = 8)	≥20 Pack-Years (n = 25)	OR	95% CI	*p*-Value
**MGMT (+), n (%)**	2 (25.0)	17 (68.0)	6.0	0.83–73.47	0.047
**MGMT (–), n (%)**	6 (75.0)	8 (32.0)			

MGMT (+), positive for MGMT promoter methylation; MGMT (−), negative for MGMT promoter methylation.

## 4. Discussion

Temozolomide is an orally administered alkylating agent that readily crosses the blood–brain barrier and is widely used in the treatment of glioblastoma [7,8]. Epigenetic silencing of the MGMT gene through promoter methylation reduces this repair capacity of O6-methylguanine DNA lesions, thereby enhancing the sensitivity of tumor cells to Temozolomide and improving therapeutic response [7,8]. Although MGMT promoter methylation is a well-established biomarker in glioblastoma [5], its association with patient clinical characteristics remains poorly understood. In this study, we demonstrated that current smoking was associated with MGMT promoter methylation in patients with glioblastoma, even after adjustment for potential confounders. According to a previous large-scale study conducted in Korea involving 9,811,768 individuals, current smokers had a higher risk of developing malignant glioma compared with never-smokers [29]. In addition, cigarette smoking has been associated with widespread and persistent genome-wide DNA methylation changes that may play a role in malignancy development [30,31]. Nicotine-derived nitrosamine ketone (NNK) is a key carcinogenic component of cigarette smoke and plays an important role in lung carcinogenesis [19]. NNK has been shown to induce DNA methyltransferase 1 (DNMT1) accumulation, leading to hypermethylation of tumor suppressor gene promoters and subsequent tumorigenesis [32]. According to a meta-analysis of 25 observational studies, hypermethylation of seven genes (CDKN2A, RASSF1, MGMT, RARB, DAPK, WIF1, and FHIT) was significantly associated with smoking behavior in patients with non–small cell lung cancer [31]. Moreover, studies in non–small cell lung carcinoma and esophageal cancer have also reported an association between smoking behavior and MGMT promoter methylation [25,33]. According to a previous study, MGMT activity was significantly decreased in the bronchial epithelial cells of current smokers compared with those of nonsmokers [34]. Taken together, previous studies and our current findings suggest a possible association between MGMT methylation and smoking. Our finding expands the current understanding of MGMT methylation beyond its prognostic and predictive role and suggests that lifestyle-related factors may be linked to epigenetic heterogeneity in glioblastoma. Although MGMT promoter methylation is the major and best-characterized mechanism of MGMT gene silencing in glioblastoma, additional epigenetic and post-transcriptional mechanisms have been described. Histone modifications, including changes in histone methylation and acetylation, can influence chromatin accessibility at the MGMT locus, potentially modulating expression [35,36]. Furthermore, several microRNAs have been reported to target MGMT mRNA and downregulate its expression via transcript degradation or translation inhibition [37,38]. Although smoking has been associated with altered expression of several microRNAs, including miR-16, miR-21, miR-146, and miR-222 [39], no smoking-related microRNA has been identified that directly targets the MGMT gene. Similarly, epigenetic modifications, including DNA promoter methylation, histone modifications, and non-coding RNAs, have been proposed to contribute to the pathogenesis of essential hypertension [20,40]. No studies to date have demonstrated an association between essential hypertension and MGMT gene promoter methylation.

However, a prior meta-analysis in non–small cell lung carcinoma reported no significant association between MGMT methylation and clinicopathologic characteristics, including age, sex, smoking status, and pathological type [41]. Similarly, previous studies in glioma patients demonstrated no association between smoking status and MGMT promoter methylation [42,43]. These findings highlight inconsistencies across studies and tumor types. Such discrepancies may be attributable to differences in tumor biology, study populations, assessment of smoking exposure, and methodological approaches used to evaluate MGMT methylation. Additionally, variations in sample size and statistical power across studies may have further contributed to the inconsistent results reported in the literature. Collectively, these inconsistencies underscore the need for tumor-specific investigations—such as the present study—to better clarify the potential role of lifestyle factors in epigenetic regulation.

In the multivariate logistic regression model, a history of hypertension showed a significant association with MGMT promoter methylation. To our knowledge, no previous studies have specifically examined the association between hypertension and MGMT promoter methylation. Therefore, the observed association in our study represents a novel finding. However, there is a lack of previously accumulated evidence; it is difficult to elucidate the association between MGMT methylation and hypertension. While previous studies have suggested associations between promoter CpG island methylation of other genes and blood pressure [20,21,44], our results represent observations from a single institution, and their significance should be interpreted with caution. Although our sample size was not sufficient to draw definitive conclusions, this result may be meaningful and warrants further investigation. Our findings may serve as a foundation for future large-scale studies aimed at clarifying the relationship between hypertension and MGMT promoter methylation and its potential biological and clinical implications. Additional studies are needed to better understand the relevance of this finding.

This study has several limitations. First, its retrospective design and single-center setting may limit the generalizability of the findings. Second, the sample size was relatively small, which may reduce statistical power and increase the risk of residual confounding. Additionally, other potential confounders influencing epigenetic regulation, such as medication use or environmental exposures, could not be fully accounted for. Nevertheless, our study identified associations between MGMT promoter methylation and both smoking and a history of hypertension. These findings raise the possibility that modification of lifestyle-related factors may influence epigenetic mechanisms involved in MGMT promoter methylation and, in turn, potentially contribute to a reduced risk of glioblastoma development driven by this carcinogenic pathway. However, further large-scale and mechanistic studies are required to clarify the causal relationships and clinical implications of these observations. Future studies are warranted to further elucidate the role of epigenetic regulation of the MGMT gene in the context of smoking and hypertension. In particular, comprehensive epigenomic analyses integrating promoter methylation, gene body methylation, histone modifications, and non-coding RNAs may help clarify whether these factors independently or synergistically influence MGMT expression. Longitudinal studies with well-defined clinical variables could also determine whether smoking status or blood pressure–related factors induce dynamic epigenetic changes in MGMT over time. From a clinical perspective, a deeper understanding of MGMT epigenetic regulation may eventually contribute to improved risk stratification or personalized therapeutic strategies, especially if modifiable environmental factors such as smoking or hypertension are shown to influence MGMT status. However, at present, the lack of direct evidence linking these factors to MGMT epigenetic alterations underscores the need for cautious interpretation and highlights an important area for future investigation.

## 5. Conclusions

This study demonstrates that MGMT promoter methylation in glioblastoma is associated with current smoking and a history of hypertension. By identifying clinical and lifestyle-related factors associated with MGMT methylation, our findings extend the current understanding of this biomarker beyond its established predictive role in treatment response. These results suggest that patient-related factors may contribute to epigenetic heterogeneity in glioblastoma. Future large-scale, prospective, and mechanistic studies are warranted to validate these associations and to determine whether modification of lifestyle or cardiovascular risk factors could influence glioblastoma development or therapeutic outcomes.

## Figures and Tables

**Table 1:** Primers used for the MGMT gene.

Methylated pair	Forward	5′-TTTCGACGTTCGTAGGTTTTCGC-3′
	Reverse	5′-GCACTCTTCCGAAAACGAAACG-3′
Unmethylated pair	Forward	5′-TTTGTGTTTTGATGTTTGTAGGTTTTTGT-3′
	Reverse	5′-AACTCCACACTCTTCCAAAAACAAAAA-3′

## Data Availability

The datasets used and/or analysed during the current study are available from the corresponding author upon reasonable request.

## References

[1] Wen P.Y., Kesari S. (2008). Malignant Gliomas in Adults. N. Engl. J. Med..

[2] Singh S., Dey D., Barik D., Mohapatra I., Kim S., Sharma M., Prasad S., Wang P., Singh A., Singh G. (2025). Glioblastoma at the Crossroads: Current Understanding and Future Therapeutic Horizons. Signal Transduct. Target. Ther..

[3] Sipos D., Raposa B.L., Freihat O., Simon M., Mekis N., Cornacchione P., Kovács Á. (2025). Glioblastoma: Clinical Presentation, Multidisciplinary Management, and Long-Term Outcomes. Cancers.

[4] Stupp R., Mason W.P., van den Bent M.J., Weller M., Fisher B., Taphoorn M.J.B., Belanger K., Brandes A.A., Marosi C., Bogdahn U. (2005). Radiotherapy plus Concomitant and Adjuvant Temozolomide for Glioblastoma. N. Engl. J. Med..

[5] Hegi M.E., Diserens A.-C., Gorlia T., Hamou M.-F., de Tribolet N., Weller M., Kros J.M., Hainfellner J.A., Mason W., Mariani L. (2005). MGMT Gene Silencing and Benefit from Temozolomide in Glioblastoma. N. Engl. J. Med..

[6] Riganti C., Salaroglio I.C., Pinzòn-Daza M.L., Caldera V., Campia I., Kopecka J., Mellai M., Annovazzi L., Couraud P.-O., Bosia A. (2014). Temozolomide Down-Regulates P-Glycoprotein in Human Blood–Brain Barrier Cells by Disrupting Wnt3 Signaling. Cell. Mol. Life Sci..

[7] Thomas A., Tanaka M., Trepel J., Reinhold W.C., Rajapakse V.N., Pommier Y. (2017). Temozolomide in the Era of Precision Medicine. Cancer Res..

[8] Barciszewska A.-M., Gurda D., Głodowicz P., Nowak S., Naskręt-Barciszewska M.Z. (2015). A New Epigenetic Mechanism of Temozolomide Action in Glioma Cells. PLoS ONE.

[9] Weller M., Van Den Bent M., Preusser M., Le Rhun E., Tonn J.C., Minniti G., Bendszus M., Balana C., Chinot O., Dirven L. (2021). EANO Guidelines on the Diagnosis and Treatment of Diffuse Gliomas of Adulthood. Nat. Rev. Clin. Oncol..

[10] Yun H.S., Kramp T.R., Palanichamy K., Tofilon P.J., Camphausen K. (2024). MGMT Inhibition Regulates Radioresponse in GBM, GSC, and Melanoma. Sci. Rep..

[11] Ganau M., Foroni R.I., Gerosa M., Zivelonghi E., Longhi M., Nicolato A. (2014). Radiosurgical Options in Neuro-Oncology: A Review on Current Tenets and Future Opportunities. Part I: Therapeutic Strategies. Tumori J..

[12] Ganau M., Foroni R.I., Gerosa M., Ricciardi G.K., Longhi M., Nicolato A. (2015). Radiosurgical Options in Neuro-Oncology: A Review on Current Tenets and Future Opportunities. Part II: Adjuvant Radiobiological Tools. Tumori J..

[13] Szylberg M., Sokal P., Śledzińska P., Bebyn M., Krajewski S., Szylberg Ł., Szylberg A., Szylberg T., Krystkiewicz K., Birski M. (2022). MGMT Promoter Methylation as a Prognostic Factor in Primary Glioblastoma: A Single-Institution Observational Study. Biomedicines.

[14] Blanc J.L., Wager M., Guilhot J., Kusy S., Bataille B., Chantereau T., Lapierre F., Larsen C.J., Karayan-Tapon L. (2004). Correlation of Clinical Features and Methylation Status of MGMT Gene Promoter in Glioblastomas. J. Neuro-Oncol..

[15] Lee M.K., Hong Y., Kim S.-Y., London S.J., Kim W.J. (2016). DNA Methylation and Smoking in Korean Adults: Epigenome-Wide Association Study. Clin. Epigenetics.

[16] Cucoreanu C., Tigu A.-B., Nistor M., Moldovan R.-C., Pralea I.-E., Iacobescu M., Iuga C.-A., Szabo R., Dindelegan G.-C., Ciuce C. (2024). Epigenetic and Molecular Alterations in Obesity: Linking CRP and DNA Methylation to Systemic Inflammation. Curr. Issues Mol. Biol..

[17] Suárez R., Chapela S.P., Álvarez-Córdova L., Bautista-Valarezo E., Sarmiento-Andrade Y., Verde L., Frias-Toral E., Sarno G. (2023). Epigenetics in Obesity and Diabetes Mellitus: New Insights. Nutrients.

[18] Long Y., Mao C., Liu S., Tao Y., Xiao D. (2024). Epigenetic Modifications in Obesity-associated Diseases. MedComm.

[19] Akopyan G., Bonavida B. (2006). Understanding Tobacco Smoke Carcinogen NNK and Lung Tumorigenesis (Review). Int. J. Oncol..

[20] Liang M. (2018). Epigenetic Mechanisms and Hypertension. Hypertension.

[21] Richard M.A., Huan T., Ligthart S., Gondalia R., Jhun M.A., Brody J.A., Irvin M.R., Marioni R., Shen J., Tsai P.-C. (2017). DNA Methylation Analysis Identifies Loci for Blood Pressure Regulation. Am. J. Hum. Genet..

[22] Louis D.N., Perry A., Wesseling P., Brat D.J., Cree I.A., Figarella-Branger D., Hawkins C., Ng H.K., Pfister S.M., Reifenberger G. (2021). The 2021 WHO Classification of Tumors of the Central Nervous System: A Summary. Neuro-Oncology.

[23] Hou Y., Sahm F. (2022). A Narrative Review of What the Neuropathologist Needs to Tell the Clinician in Neuro-Oncology Practice Concerning WHO CNS5. Glioma.

[24] WHO Expert Consultation (2004). Appropriate Body-Mass Index for Asian Populations and Its Implications for Policy and Intervention Strategies. Lancet.

[25] Liu Y., Lan Q., Siegfried J.M., Luketich J.D., Keohavong P. (2006). Aberrant Promoter Methylation of P16 and MGMT Genes in Lung Tumors from Smoking and Never-Smoking Lung Cancer Patients. Neoplasia.

[26] Christmann M., Kaina B. (2012). O6-Methylguanine-DNA Methyltransferase (MGMT): Impact on Cancer Risk in Response to Tobacco Smoke. Mutat. Res. —Fundam. Mol. Mech. Mutagen..

[27] Matsuda S., Mafune A., Kohda N., Hama T., Urashima M. (2020). Associations among Smoking, MGMT Hypermethylation, TP53-Mutations, and Relapse in Head and Neck Squamous Cell Carcinoma. PLoS ONE.

[28] Neumann T., Rasmussen M., Heitmann B., Tønnesen H. (2013). Gold Standard Program for Heavy Smokers in a Real-Life Setting. Int. J. Environ. Res. Public Health.

[29] Ahn S., Han K.-D., Park Y.-M., Bae J.M., Kim S.U., Jeun S.-S., Yang S.H. (2020). Cigarette Smoking Is Associated with Increased Risk of Malignant Gliomas: A Nationwide Population-Based Cohort Study. Cancers.

[30] Joehanes R., Just A.C., Marioni R.E., Pilling L.C., Reynolds L.M., Mandaviya P.R., Guan W., Xu T., Elks C.E., Aslibekyan S. (2016). Epigenetic Signatures of Cigarette Smoking. Circ. Cardiovasc. Genet..

[31] Huang T., Chen X., Hong Q., Deng Z., Ma H., Xin Y., Fang Y., Ye H., Wang R., Zhang C. (2015). Meta-Analyses of Gene Methylation and Smoking Behavior in Non-Small Cell Lung Cancer Patients. Sci. Rep..

[32] Lin R.-K., Hsieh Y.-S., Lin P., Hsu H.-S., Chen C.-Y., Tang Y.-A., Lee C.-F., Wang Y.-C. (2010). The Tobacco-Specific Carcinogen NNK Induces DNA Methyltransferase 1 Accumulation and Tumor Suppressor Gene Hypermethylation in Mice and Lung Cancer Patients. J. Clin. Investig..

[33] Das M., Sharma S.K., Sekhon G.S., Saikia B.J., Mahanta J., Phukan R.K. (2014). Promoter Methylation of MGMT Gene in Serum of Patients with Esophageal Squamous Cell Carcinoma in North East India. Asian Pac. J. Cancer Prev..

[34] Povey A.C., O’Donnell P., Barber P., Watson M., Margison G.P., Koref M.F.S. (2006). Smoking Is Associated with a Decrease of O6 -alkylguanine-DNA Alkyltransferase Activity in Bronchial Epithelial Cells. Int. J. Cancer.

[35] Fang C., Zhang G., Ye S., Tian S., Li H., Zuo F., Wan J., Cai H. (2025). Regulatory Mechanisms of O6-Methylguanine Methyltransferase Expression in Glioma Cells. Sci. Prog..

[36] Nakagawachi T., Soejima H., Urano T., Zhao W., Higashimoto K., Satoh Y., Matsukura S., Kudo S., Kitajima Y., Harada H. (2003). Silencing Effect of CpG Island Hypermethylation and Histone Modifications on O6-Methylguanine-DNA Methyltransferase (MGMT) Gene Expression in Human Cancer. Oncogene.

[37] Kreth S., Limbeck E., Hinske L.C., Schütz S.V., Thon N., Hoefig K., Egensperger R., Kreth F.W. (2013). In Human Glioblastomas Transcript Elongation by Alternative Polyadenylation and miRNA Targeting Is a Potent Mechanism of MGMT Silencing. Acta Neuropathol..

[38] Khalil S., Fabbri E., Santangelo A., Bezzerri V., Cantù C., Gennaro G.D., Finotti A., Ghimenton C., Eccher A., Dechecchi M. (2016). miRNA Array Screening Reveals Cooperative MGMT-Regulation between miR-181d-5p and miR-409-3p in Glioblastoma. Oncotarget.

[39] Kaur G., Begum R., Thota S., Batra S. (2019). A Systematic Review of Smoking-Related Epigenetic Alterations. Arch. Toxicol..

[40] Wise I., Charchar F. (2016). Epigenetic Modifications in Essential Hypertension. Int. J. Mol. Sci..

[41] Chen L., Wang Y., Liu F., Xu L., Peng F., Zhao N., Fu B., Zhu Z., Shi Y., Liu J. (2018). A Systematic Review and Meta-Analysis: Association between MGMT Hypermethylation and the Clinicopathological Characteristics of Non-Small-Cell Lung Carcinoma. Sci. Rep..

[42] Guan Y., Li Y., Li Y. (2019). The Change of MGMT Gene Expression in Glioma Patients Was Affected by Methylation Regulation and in the Treatment of Alkylation Agent. Int. J. Clin. Exp. Med..

[43] Malueka R.G., Hartanto R.A., Alethea M., Sianipar C.M., Wicaksono A.S., Basuki E., Dananjoyo K., Asmedi A., Dwianingsih E.K. (2022). Associations among Smoking, IDH Mutations, MGMT Promoter Methylation, and Grading in Glioma: A Cross-Sectional Study. F1000Research.

[44] Kato N., Loh M., Takeuchi F., Verweij N., Wang X., Zhang W., BIOS-Consortium, CARDIo GRAMplusCD, LifeLines Cohort Study, The InterAct Consortium (2015). Trans-Ancestry Genome-Wide Association Study Identifies 12 Genetic Loci Influencing Blood Pressure and Implicates a Role for DNA Methylation. Nat. Genet..

